# Sharing an environment with sick conspecifics alters odors of healthy animals

**DOI:** 10.1038/s41598-018-32619-4

**Published:** 2018-09-24

**Authors:** Stephanie S. Gervasi, Maryanne Opiekun, Talia Martin, Gary K. Beauchamp, Bruce A. Kimball

**Affiliations:** 1 Monell Chemical Senses Center, 3500 Market Street, Philadelphia, PA 19104 USA; 2 USDA-APHIS-WS-NWRC, 3500 Market Street, Philadelphia, PA 19104 USA

## Abstract

Body odors change with health status and the odors of sick animals can induce avoidance behaviors in healthy conspecifics. Exposure to sickness odors might also alter the physiology of healthy conspecifics and modify the odors they produce. We hypothesized that exposure to odors of sick (but non-infectious) animals would alter the odors of healthy cagemates. To induce sickness, we injected mice with a bacterial endotoxin, lipopolysaccharide. We used behavioral odor discrimination assays and analytical chemistry techniques followed by predictive classification modeling to ask about differences in volatile odorants produced by two types of healthy mice: those cohoused with healthy conspecifics and those cohoused with sick conspecifics. Mice trained in Y-maze behavioral assays to discriminate between the odors of healthy versus sick mice also discriminated between the odors of healthy mice cohoused with sick conspecifics and odors of healthy mice cohoused with healthy conspecifics. Chemical analyses paired with statistical modeling revealed a parallel phenomenon. Urine volatiles of healthy mice cohoused with sick partners were more likely to be classified as those of sick rather than healthy mice based on discriminant model predictions. Sickness-related odors could have cascading effects on neuroendocrine or immune responses of healthy conspecifics, and could affect individual behaviors, social dynamics, and pathogen spread.

## Introduction

Living in groups can increase access to resources such as food, shelter, and mates^1^, but can also impose direct costs on individuals, such as increased competition, greater conspicuousness to predators, and higher risk of infections transmitted from conspecifics^1–5^. The ability to identify and respond to indicators of health status is therefore critical in groups. Many animals, including humans, have evolved sophisticated sensory mechanisms to detect the risk of contagion in the social environment^6–10^. Visual, auditory, somatosensory, and olfactory cues are processed by the central nervous system and can orchestrate physiological and behavioral responses, enabling an organism to optimize survival and reproductive success under potentially adverse circumstances^6,8,11^.

Chemical cues are key mediators of two-way communication in social animals^6,8,12–14^, and convey fixed characteristics of individuals such as sex, age, and relatedness, as well as dynamic traits, such as physical condition, social dominance status, and health^6,13–16^. Body odor may be among the most salient indicators of risk of contagion in an individual’s social environment^6,16,17^, particularly if visual or other types of pathological signs of infection or illness are absent, attenuated, or socially suppressed^18^. A large body of literature has shown that chronic disease, pathogenic and parasitic infection, inflammation, and vaccination can alter body odors^15,19–29^. The consequences of such odor changes can be context-dependent^20,30–39^, but experimental studies with rodents and humans have generally shown that healthy individuals limit their investigation of and rate as more aversive the odors of sick or infected, compared to healthy individuals^10,16,17,22,27,40^. Several studies have specifically examined behavioral responses of healthy rodents to the odors of conspecifics injected with the non-replicating bacterial endotoxin, lipopolysaccharide (LPS)^20,32,35,40^. Notably, most of these behavioral assessments of responses to the odors of LPS-injected individuals have involved presentation of odor stimuli (e.g., live animals or their urine or bedding material) within 4 hours of injection^20,32,35,40^. This early time point following LPS injection is associated with robust pro-inflammatory immune responses, increased body temperature (fever), and the expression of sickness behaviors such as lethargy, anorexia, and self-isolation^23,41–43^. It is unknown whether avoidance or constrained social investigation of sick animals and their odors by healthy conspecifics persists beyond early time points associated with inflammation. However, evidence from behavioral discrimination assays suggests that odor changes associated with LPS injection persist for at least two weeks, and possibly more than 24 days, following a single injection^44^.

The physiological effects of exposure to the odors of sick or infected conspecifics have also been examined, though less extensively than behavioral effects. Alves and colleagues showed that exposure to the odors of tumor-bearing conspecifics reduced the intensity of some aspects of innate immunity in healthy cagemates^45,46^. In another study, Kavaliers and Colwell showed that exposure to the scent of male mice infected with a protozoan parasite caused increased analgesic responses and associated elevation of pain tolerance in healthy female mice^25,47^. Taken together, the behavioral and physiological effects of exposure to odors indicative of health status could have complex feedbacks on ecological and epidemiological dynamics^42^. For example, altered patterns of physical contact and changes in immunological and other physiological traits among healthy animals could have cascading effects on group dynamics, social network structure, and spatiotemporal patterns of disease spread^48^.

**Figure 1:**
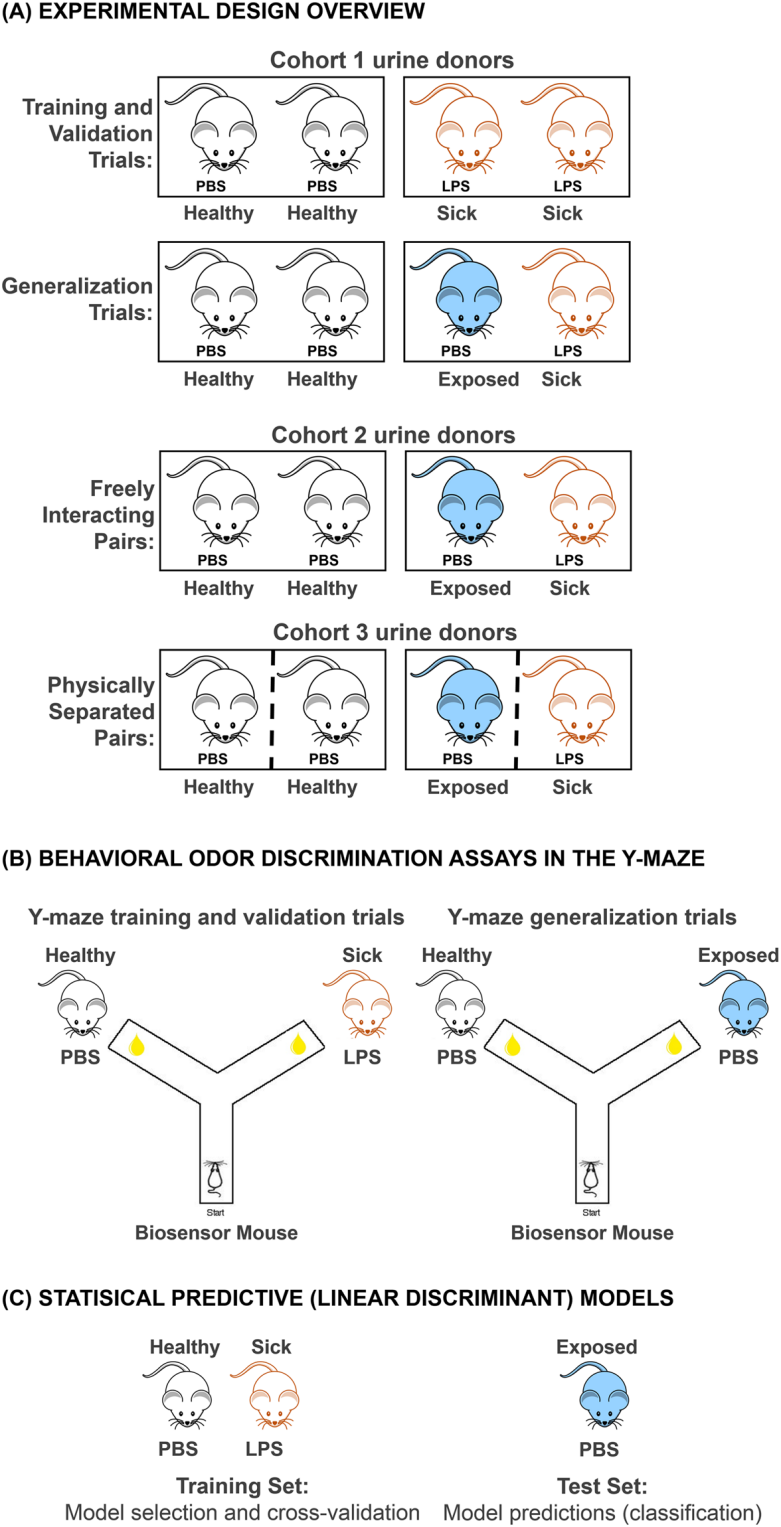
Experimental design and testing overview. Our experimental design involved three separate mouse cohorts, cohort 1, 2, and 3 (outlined in **A**), which provided urine stimuli for behavioral (**B**) or chemical (**C**) analyses. Urine stimuli from cohort 1 mice were used in the behavioral odor discrimination assays employing the Y-maze. Within cohort 1, separate sub-groups of urine donors provided stimuli for training, validation, and generalization trials (for full descriptions of trial types see the “Biosensor mouse care and testing in the Y-maze” section of our Methods). For Y-maze training and validation trials (**B**, left) urine donor mice were pair housed and could freely interact with identically-treated cagemates, who were either sick (LPS-injected; orange outline) or healthy (PBS-injected; black outline). These mice are referred to as “sick” and “healthy”, respectively. Urine donor mice providing stimuli for Y-maze generalization trials (**B**, right) were also pair housed and could freely interact. However, the mice providing urine stimuli for Y-maze generalizations consisted of healthy mice who were pair housed with either sick (LPS-injected) or with healthy (PBS-injected) partners (**B**, right). Healthy mice housed with healthy partners are referred to as “healthy” (black outline), while healthy mice housed with sick partners are referred to as “exposed” (blue outline and blue fill). LPS-injected mice are referred to as “sick” (orange outline). Mice in cohort 2 and 3 provided urine stimuli for GC-MS analysis and statistical predictive modeling (**C**). We use identical nomenclature to refer to mice as healthy, sick, or exposed, in cohorts 2 and 3 depending treatment and identity of cagemates. However, mice in cohorts 2 and 3 differed with respect to cohousing setup. Specifically, mice in cohort 2 could freely interact (similarly to cohort 1 mice) while mice in cohort 3 occupied the same cage but were physically separated by a semi-permanent partition which permitted odor transfer throughout the cage. Linear discriminant models were used to describe the differences in volatiles of sick versus healthy mice (**C**, left). These models, describing sick and healthy mice, were used to make subsequent predictions about the identity of exposed mouse samples as more similar to those of sick or to healthy mice (**C**, right). Mouse drawings used to create Fig. 1 were obtained from WPClipart: https://www.wpclipart.com/animals/M/mouse/mouse_2/mouse_clip_outline.png.html. WPClipart images are an online collection of free artwork collected and edited by Paul Sherman.

We asked whether exposure to the odors of sick but non-infectious conspecifics could subsequently affect the odors of healthy animals. To induce sickness, we injected mice with LPS from *Esherichia coli* (*E*. *coli*). Healthy mice were injected with a volume-matched placebo, consisting of a sterile phosphate-buffered saline (PBS) solution, which does not induce sickness behaviors or activate inflammatory immune responses, but which controlled for the effects of handling and the injection procedure across experimental animals. We hypothesized that the odors of healthy/PBS-injected mice housed together with other healthy/PBS-injected mice (referred to as “healthy” mice; Fig. 1A) would be distinct from odors of healthy/PBS-injected mice cohoused with “sick”/LPS-injected partners (referred to as “exposed” mice; Fig. 1A). More specifically, we predicted that the odors of exposed mice would be more similar to the odors of sick mice than to the odors of healthy mice. Healthy mouse odors might change in the presence of sick conspecifics if sensory cues indicating a possible risk of a transmission event activate immunological defenses^49^ or induce a neuroendocrine stress response to a perceived threat to homeostasis.

To experimentally test our hypothesis we employed behavioral, chemical, and statistical approaches which involved biosensor animal odor discrimination assays in a Y-maze setup, dynamic headspace analysis employing gas chromatography and mass spectrometry, and statistical predictive models based on urinary volatile odorants (Fig. 1 and Supplementary Fig. S1). Our parallel approaches were intended to be complementary. Both behavioral and statistical approaches employed training and validation procedures followed by testing procedures to arrive at predictions about the odors of healthy partners of sick mice (Fig. 1B,C). The primary outcome of interest in both approaches was how novel samples from these exposed mice were classified by biosensor animals or statistical models (i.e., as more similar to sick or more similar to healthy mouse samples). To ascertain whether urinary volatile changes were a function of direct physical or environmental transfer we used two different housing setups. Specifically, we allowed one experimental cohort of mice to freely interact in cages, while another cohort of mice was cohoused but physically separated with semi-permeable cage partitions. Cage partitions contained 8 small (0.5 mm) holes and precluded direct physical contact between mice and their bedding materials while permitting odorant transfer throughout the cage (Fig. 1A).

## Results

### Effects of LPS-injections

Within a few hours after injection with the endotoxin LPS, mice in all three cohorts (Fig. 1A) demonstrated typical sickness behaviors^23,41,43^ such as lethargy (reduced activity) and weight loss. Sick, LPS-injected mice lost body weight by 3.00 ± 0.14 g (mean ± SE, urine donor cohorts 1–3; representing 13–14% of initial body weight) within the first 24 h after receiving LPS injection. No mortality was observed in mouse cohort 1 or 2 (Fig. 1A). However, within the physically separated cohort of mice (cohort 3, Fig. 1A) two LPS-injected mice died (one individual 4 days after injection, and one individual 5 days after injection). Healthy/PBS-injected mice tended to gain body weight within 24 h of receiving PBS injections, and this was the case whether healthy mice were housed with other healthy/PBS-injected mice or with sick/LPS-injected mice. Specifically, across cohorts 1–3, healthy mice displayed a gain of 0.11 ± 0.09 g and exposed mice displayed a gain of 0.06 ± 0.08 g.

### Behavioral odor discrimination assays in the Y-maze (cohort 1)

**Figure 2:**
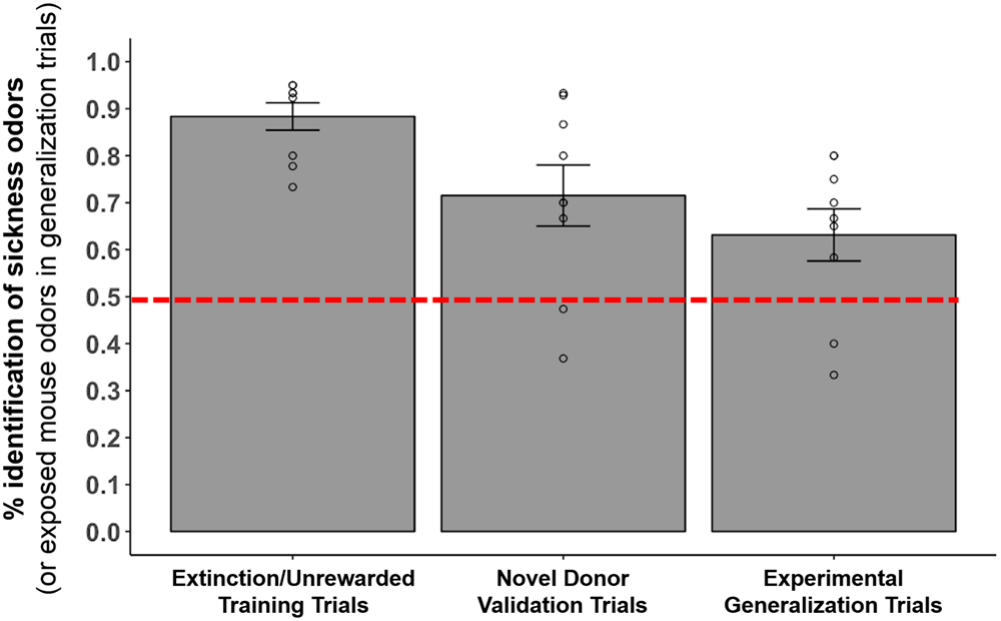
Biosensor mouse performance in Y-maze odor discrimination assays. Biosensor mice (N = 9) initially underwent reward-based training to identify the arm of the Y-maze containing urine odors from sick/LPS-injected versus healthy/PBS-injected mice, and were reinforced to (*i*.*e*., received a reward for choosing) odors of sick conspecifics. Here, we display performance of biosensors in extinction/unrewarded training trials, novel donor validation trials, and experimental generalization trials (for full descriptions of trial types see the “Biosensor mouse care and testing in the Y-maze” section of our Methods). For extinction and validation trials, bar height corresponds to the mean percentage of trials in which biosensor mice correctly identified the arm of the Y-maze containing odors of sick conspecifics. For generalization trials, bar height corresponds to the mean percentage of trials in which the same biosensor mice chose the arm of the Y-maze containing odors of exposed, versus healthy, conspecifics. Error bars represent +/−1 SEM and individual biosensor performance is denoted with open circles. The two biosensor mice below the 50% line were not the same individuals in validation and generalization trials. All nine circles corresponding to individual biosensor responses may not be visible because of overlapping symbols/biosensor performance. On average, the responses of biosensor mice within each trial type differed significantly from 50/50, or random choice of the odors/arms of the Y-maze (independent exact binomial tests for each trial type were all P < 0.01).

In rewarded experimental training trials biosensor mice (N = 9) correctly identified the Y-maze arm that contained urine from sick mice versus urine from healthy mice in 90% of the trials, on average (1466/1619 trials). This 90% success rate in training trials differed significantly from 50% chance, or random choice of one or the other of the two Y-maze arms (p < 0.0001, exact binomial test). Similarly, in extinction trials (i.e., unrewarded training trials), these same biosensor mice correctly identified the Y-maze arm containing urine from sick versus healthy mice in 90% of the trials (272/302 trials), and this average response was also significantly different from 50/50 chance in the 2-choice Y-maze behavioral assay (p < 0.0001, exact binomial tests, Fig. 2). In crucial double blind unrewarded experimental Y-maze validation trials with novel donor mice (urine samples from sick and healthy mice never before encountered by the trained mice), the same nine biosensor animals correctly chose the maze arm associated with urine of novel sick mice compared to novel healthy mice in 70% of the trials (108/155 trials), which was significantly greater than random choice (p < 0.0001, exact binomial test, Fig. 2). Previous work has shown that despite the fact that biosensor mice are presented with odors from identically-treated mice in both Y-maze training and validations trials, performance often decreases incrementally when mice are presented with novel urine donors in validations^23^. This suggests that mice learn some features of individual identity as well as treatment level effects in training^23,50^. Finally, in double blind unrewarded generalization trials designed to directly test our hypothesis, biosensor mice chose the maze arm associated with urine of novel exposed mice (healthy/PBS-injected mice housed with sick/LPS-injected mice) versus the Y-maze arm containing urine of novel healthy mice (healthy/PBS-injected mice housed with healthy/PBS-injected mice) in 63% of trials (92/145 trials), which was also significantly greater than random selection of Y-maze arms in the behavioral assay (p = 0.001, exact binomial test, Fig. 2). Responses of biosensor mice were grouped within session type (extinctions, validations, generalizations) since a preliminary regression analysis revealed no predictive effect of time (day of testing effect; X^2^_3_ = 1.2; P = 0.75), individual biosensor mouse performance (ID effect; X^2^_8_ = 0.94; P = 0.99), or trial type (extinction trials within validation sessions versus extinctions trials within generalization sessions; X^2^_1 = _0.51; P = 0.47) on responses of biosesnsor mice in the Y-maze behavioral assay.

### Statistical predictive modeling based on urine odorants identified by GC-MS

#### Cohoused and freely interacting mice (cohort 2)

We identified 68 peaks in processed chromatograms describing volatiles contained in mouse urine. Using stepwise feature selection, we identified a three-peak model which met our *a priori* model selection criteria with a squared canonical correlation value of 0.61 or 61% and this model could not be further improved by removal or inclusion of additional peaks. Model selection and linear discriminant analysis were performed on our training data set that included urine samples from sick/LPS-injected and healthy/PBS-injected mice, only (Fig. 1C). Our three-peak model was 89% accurate in classifying sick and healthy mouse samples in leave-one-out cross-validation. Specifically, 7/10 healthy mouse samples and 17/17 sick mouse samples were correctly classified in the cross-validation procedure (Supplementary Fig. S2). The individual volatiles represented in the three-peak model to accurately discriminate sick from healthy mouse samples were identified as 2-acetyl-2-thiazoline, decanal, and dimethyl sulfone based on NIST spectral library matching and relative retention times^24,51–53^ (Supplementary Table S1). Sick mice tended to have higher levels of decanal and dimethyl sulfone compared to healthy mice, although post-hoc pairwise comparisons of these compound levels between treatments were non-signficant (Supplementary Table S2A). Conversely, healthy mice tended to have signficantly higher levels of 2-acetyl-2-thiazoline than sick mice (Supplementary Table S2A). Predictions about sample classification applied to the test data set (*i*.*e*., classification predictions pertaining to the identity of exposed mouse urine samples) and based on the same linear discriminant model fit to the training data set of sick and healthy mice, resulted in the classification of 12 out of 16 exposed mice as sick and 4/16 exposed mice as healthy. The difference in mean posterior probability associated with sick versus healthy classification of the exposed mouse samples was statistically significant (W = 65, P = 0.0189). In other words, based on the three peaks selected for our sick versus healthy discriminant model, 75% of the exposed mouse urine samples were identified as more chemically similar to sick mouse samples than to healthy mouse samples, and there was a significant difference in the predicted classification of exposed mice as sick versus healthy. Mean processed chromatograms for cohort 2 mice are provided in Supplementary Fig. S3.

#### Cohoused but physically separated mice (cohort 3)

Because of differences in housing conditions and timing of experiments between cohorts 2 and 3 (Fig. 1A), we built a second discriminant model for the cohoused but physically separated cohort of mice. However, we applied an identical process of stepwise feature/model selection, discriminant analysis, and cross-validation applied to our training data set (consisting of sick/LPS-injected and healthy/PBS-injected mice), followed by predictive classification of a novel test set of exposed mouse urine samples (Fig. 1C). Thus, the same 68 peaks were chosen as a starting point for model selection for cohort 3. We identified a final model, which again happened to include a total of three peaks, and met our *a prioiri* model selection criteria. This three-peak model had a squared canonical correlation of 0.86 or 86% and was 100% accurate in leave-one-out cross-validation. Specifically, 6/6 healthy mouse urine samples and 5/5 sick mouse urine samples were correctly classified in leave-one-out cross-validation (Supplementary Fig. S4). The three compounds that discriminated between healthy and sick mice in the physically separated cohort of mice were 6-methyl-3-heptanone, 4-methyl-6-hepten-3-one, and dehydro-*exo*-brevicomin. The latter two compounds tended to be elevated in sick mice compared to healthy mice, while 6-methyl-heptanone tended to be elevated in healthy versus sick mice. Post-hoc pairwise comparisons, which were not part of our primary and intended pattern recognition approach, suggested significant treatment-level differences in 4-methyl-6-hepten-3-one, only (Supplementary Table S2B). Discriminant-model-based predictions about the test data set samples collected from exposed mice resulted in classification of 6 out of 7 exposed mice as sick and 1/7 exposed mouse as healthy. The difference in mean posterior probability associated with sick versus healthy classification of exposed mouse urine samples was statistically significant (W = 1, P = 0.003). In other words, based on the three peaks selected to discriminate between sick and healthy mice in the training data set, 86% of the exposed mouse urine samples in the test data set were identified as more chemically similar to those of sick compared to healthy mice, and there was a significant difference in the predicted classification of exposed mice as sick versus healthy. Mean processed chromatograms for mice in cohort 3 are provided in Supplementary Fig. S5.

#### Across cohort model evaluation

Although mouse cohort 2 and 3 were housed under different conditions, we additionally investigated the performance of our discriminant models in classifying sick and healthy mouse samples across mouse cohorts. The three-peak model originally selected to discriminate between sick and healthy mice within cohort 3 correctly classified 13/17, or 76% of sick mice in cohort 2 as sick, but correctly classified only 3/10 healthy mice in cohort 2 as healthy. Conversely, the three-peak model originally selected to discriminate between sick and healthy mice within cohort 2 correctly classified only 2/5 sick and 2/6 healthy mice in in cohort 3. This suggests strong within-cohort effects and high among-cohort variation, due to housing conditions and possibly other factors, discussed below. Because of poor model performance in across-cohort classification, we made all hypothesis-based predictions about exposed mouse samples within cohort 2 or 3, as described in the results, above.

## Discussion

We employed two complementary methods in parallel to explore the hypothesis that exposure to sick but non-infectious conspecifics alters the odors produced by healthy mice. Both Y-maze behavioral discrimination assays employing biosensor mice and statistical predictive classification models based on volatile odorants suggested a common phenomenon: odors of healthy mice living with sick conspecifics more closely resembled the odors of sick (versus healthy) mice. Although our two methodological approaches differed, both had a common goal of predicting health status of individual mice based on their urinary volatiles. Both behavioral and statistical approaches employed training and validation followed by testing procedures to arrive at predictions about the body odors of healthy partners of sick mice (e.g., exposed mice).

First, biosensor mice were trained via positive reinforcement to discriminate between the urine odors of sick/LPS-injected and healthy/PBS-injected mice (Fig. 1A,B, Cohort 1 urine donors). Classification accuracy of biosensor mice was high (70%) in validation trials with novel (never before seen) sick and healthy urine donors (Fig. 1A,B, Cohort 1 urine donors). Testing in experimental generalization trials was the most critical aspect of our procedure, since this allowed for a direct test of our hypothesis about the classification of novel urines from exposed mice. In double blind unrewarded trials, biosensor mice generalized what they learned about urinary volatiles of sick mice during training to the volatiles of healthy mice who were housed with sick conspecifics (i.e., exposed mice; Fig. 1B). In other words, biosensor mouse behavior in the Y-maze indicated that the odors of exposed mice were more similar to the odors of LPS-injected, compared to PBS-injected mice. The value of the Y-maze paradigm for generalization-based testing has been demonstrated in numerous studies^19,23,41,44,50^.

Following behavioral discrimination trials in the Y-maze, we next employed predictive classification modeling based on the volatiles identified with GC-MS. Models were selected and initially fit with a training data set, consisting of chromatographic peak responses for urine volatiles of sick/LPS-injected and healthy/PBS-injected mice (Fig. 1C). Model accuracy in classifying (or discriminating between) sick versus healthy mouse samples was high (89% and 100% for mouse cohort 2 and 3, respectively) as determined by leave-one-out cross-validation. Notably, these modeling procedures applied to chemical (GC-MS) data were carried out across two differently-housed cohorts of mice (Cohort 2 and 3 urine donors, Fig. 1A,C). In one cohort, mice could physically interact (cohort 2). In another cohort, mice were also cohoused, but were physically separated by semi-permeable cage partitions, which allowed odorants to pass throughout the cage (cohort 3; validation of odor transfer in partitioned cages shown in Supplementary Fig. S6). These two different housing conditions were employed to investigate whether any alterations in urinary volatiles in exposed mice might be due to environmental transfer of odorants (e.g., via coprophagy or direct transfer of odorants via bodily contact) onto healthy animals through social interactions in a shared cage. Since the volatiles of exposed mice were more likely to be predictively classified as sick versus healthy mouse volatiles within both cohorts of mice, we conclude that alteration of healthy mouse urine volatiles is unlikely to be a result of direct physical odorant transfer. Rather, we suggest that physiological changes were induced in mice exposed to the odors of sick cagemates, and these physiological changes may have shared some resemblance to the physiological alterations induced by LPS injections in sick mice.

While the major goal of this work was to use trained biosensor mice and statistical models to classify healthy animals exposed to sickness odors as more similar to sick or healthy conspecifics, several individual odorants were identified that may have diagnostic significance. The most striking odorant commonality in this study and in similar studies previously published (42 and 45) is the apparent centrality of changes in compounds that mice use in social communication. All three compounds that were selected to discriminate between sick and healthy mice in cohort 3 are thought to be related to putative mouse pheromones. These compounds included dehydro-exobrevicomin (DHB), 4-methyl-6-hepten-3-one, and 6-methyl-3-heptanone. Specifically, DHB responses were higher in urine of sick mice compared to healthy mice. The chemical DHB, produced by male mice, has been proposed to be involved in oestrus synchronization and puberty acceleration in female mice, as well as inter-male aggression^54,55^. Two previously published studies comparing odors of LPS- versus PBS-injected mice also identified DHB as a key predictor of health status^41,44^. Consistent with our study, work by Millet *et al*.^41^ and Kimball *et al*.^44^ showed an increase in DHB in sick/LPS-injected compared to healthy/PBS-injected mice (including unpublished data, Kimball *et al*.^44^). Support for LPS-induced changes in DHB across experimental studies also suggests that this compound could serve as a potential biomarker for LPS-induced sickness. Both ketones included in predictive classification models for cohort 3 may be related to 6-hydroxy-6-methyl-3-heptanone (HMH), another putative male mouse pheromone involved in acceleration of puberty in female mice^55^. HMH appeared as a top predictor of health status in the Kimball *et al*. study^44^; specifically, levels of this pheromone were lower in LPS-injected compared to PBS-injected mice which is consistent with the trend we observed for 6-methyl-3-heptanone. Interestingly, a previous behavioral study showed that odors of male mice infected with influenza virus were less attractive to, but not strongly avoided by female mice^28^. Our chemical data suggests that sickness induced by LPS alters compounds mice use in inter- and intra-sexual communication.

The compound 2-acetyl-2-thiazoline was predictive of health status in the cohort of freely interacting mice (cohort 2) and may be involved in production of 2-*sec*-butyl-4,5-dihydrothiazole (SBT). SBT is a mouse pheromone that acts as a conspecific warning signal and also facilitates inter-male aggression and the acceleration of puberty in female mice^54–56^. Decanal, which was more abundant in sick compared to healthy mice in cohort 2, is a known constituent of C57BL/6 J mouse urine and is hypothesized to play a role in conspecific recognition^57^. Dimethyl sulfone, which was predictive of health status in cohort 2, has been linked to the intestinal microbiota in humans^58^. In other studies, this compound has been shown to have anti-inflammatory effects^59,60^. The appearance of dimethyl sulfone in our predictive models could indicate a possible compensatory response to inflammation or a change in gut microbes following LPS injections.

Compound identification might inform future mechanism-based studies about metabolic and immunological pathways involved in odor changes across different health conditions. However, explicit interpretation of the roles of specific odorants and/or direct comparisons with other studies should proceed with caution. First and foremost, no attempt was made in this study to describe “global” sickness odorants across studies and cohorts. Rather, just as unique panels of trained biosensor mice have been employed for different studies in our laboratory, unique discriminant models were constructed for each cohort in this study. Groups of compounds/peaks selected using our pattern recognition approach to accurately discriminate between sick and healthy mice in different studies^41,44^ and different cohorts of the present study are likely influenced by variability within the mice themselves (e.g. cohort/litter effects) or to subtle but biologically relevant external environmental or investigator (e.g., handling) effects. Within- or among-day effects of urine collection as well as sampling variation across relatively small sample sizes could also underlie differences observed across cohorts and previous studies. We also suggest that pairwise comparisons for individual compounds given in Supplementary Table S2A,B should be interpreted with caution since they do not reflect the primary goal of our analysis of chemical data, which was instead based on pre-determined model selection and classification procedures, followed by hypothesis-based predictions about our test data set of exposed mouse urine samples.

We suspect that social housing condition interacted with treatment effects in this study, leading to variation in the compounds that best differentiated health status between cohorts and resulting low across- (but high within-) cohort model performance (Supplementary Table S3). Application of within-cohort discriminant models to differently housed mouse cohorts resulted in poor classification of sick and healthy mice. We often observed freely interacting pairs of mice in cohort 2 nesting together, and this was observed both before and after injections with LPS or PBS, which was surprising in light of previous work suggesting reduced social investigation and increased avoidance-like behaviors of healthy animals exposed to LPS-injected conspecifics or their odors^32,40^. Thermoregulation could have differentially affected odors produced by mouse cohorts, since co-nesting was not possible in physically separated mice in cohort 3. Physical separation of mice without complete blocking of visual, auditory, and odor cues in cohort 3 conditions might have also affected levels of certain hormones (e.g., testosterone or corticosterone) and/or dominance or scent-marking behaviors of male mice in our study, leading to differences in volatiles selected in discriminant model building procedures. Because there were small (0.5 mm) holes present in cage partitions used in the experimental setup for cohort 3 mice, we cannot completely rule out the possibility that some small particles or microorganisms were transferred between animals that shared a cage. Though not explored in this study, the transfer of commensal microbes from sick mice to healthy mice could have contributed to altered physiology^61^ and/or altered odors of cohoused healthy (exposed) mice. Such an effect would potentially be more pronounced in the freely interacting compared to physically separated mice.

A logical next step in this research area should involve the investigation of additional physiological and behavioral changes in healthy partners of sick animals. That is, future studies could explore other traits that are sensitive to exposure to volatile sickness cues. It is known that exposure to the odors of conspecifics and interspecifics (e.g., predators) can drastically alter rodent physiology including neuroendocrine and immune responses^6,16,62–64^, exposure to odors of tumor-bearing mice reduces innate immune responses in healthy conspecifics^45,46^, and exposure to the scent of pathogen-infected male mice can induce opioid and nonopioid mediated analgesia in female mice^25,47^. Studies in humans have shown that body odors of LPS-injected subjects are ranked as more aversive and less attractive compared to the body odors of placebo/PBS-injected subjects^10,22^. Thus, exposure to odors of sick conspecifics, even in the absence of actual parasitic or pathogenic transmission, might condition or prime aspects of behavior or physiology (e.g., immunity) to reduce the risk of contagion and/or alter susceptibility and resistance to impending infection.

In addition to potential effects on immunity and disease resistance/susceptibility, exposure to the odors of sick conspecifics might initiate a neuroendocrine response, as would occur with exposure to an environmental stressor that perturbs an organism from homeostasis. To test the hypothesis that sickness odors act as stressors and elicit subsequent physiological responses, investigators might examine changes in levels of adrenocorticotropic hormone, corticosterone, or catecholamines. Altered activity of the hypothalamus-pituitary-adrenal axis might be seen in circulating blood or at the receptor level in the brain or other tissues. Animals with elevated stress hormone levels might also have odorant profiles that resemble exposed mice and/or sick mice. In this study, our statistical models were not explicitly used to discriminate among healthy, sick, and exposed mice. Instead, we asked about resemblance of exposed mouse odors to the odors of healthy or sick mice. Future work might focus on discriminating among groups of stressed, immune-stimulated, and infected animals with behavioral or chemical assays.

## Conclusions

Our study revealed a novel aspect of animal odors and odorants produced by simulated infection with the bacterial endotoxin, lipopolysaccharide (LPS). Previous studies have demonstrated that sick animals produce odors that are distinct from those of healthy animals^6,15–17^. Here, we showed that mice that are exposed to odors of sick animals take on aspects of the sick animals’ odors and odorant profiles. Although not explored in the current report, this result could have important implications for disease dynamics in animal, perhaps including human, populations.

Volatile compounds indicative of health status could serve as important biomarkers for the detection and diagnosis of disease in a clinical and field setting^15,21,23,24^. Behavioral and chemistry-based methods could further inform the development of novel olfactory-based disease detection techniques, such electronic sensory devices. Ultimately, a more universal study aimed at identifying consistent volatile biomarkers of health and disease might involve a meta-analytical or a prediction-based approach that explicitly combines data across multiple studies and even across animal taxa.

## Methods

### Animal Care and Use Statement

All methods were approved by and carried out in accordance with Monell Institutional Animal Care and Use Committee (IACUC) guidelines and regulations under approved protocol #1176.

### Cohort 1: Behavioral odor discrimination assays in the Y-maze

#### Urine donor care

Thirty-six four-week old male C57BL/6 J mice were obtained commercially, from Jackson Laboratories (Bar Harbor, ME). Upon arrival to the Monell Chemical Senses Center animal facility, mice were pair housed in 11.5 × 8-inch plastic shoebox containers with approximately 30 grams of wood shaving bedding per cage (cedar chip laboratory bedding, Northeastern Products Corporation, Warrensburg, NY). Mice had *ad libitum* access to clean drinking water and chow (Teklad Rodent Diet 8604, Harlan, Madison, WI) and full cage changes were performed weekly. Ambient room temperature was controlled at ~74–76° F and mice were housed on a 12:12 light/dark cycle (lights on at 7:00 am and off at 7:00 pm). Animals were checked twice daily for general health and condition. Mice were acclimated to the animal housing facility for ~2 weeks prior to the start of experimental protocols and were therefore ~6–7 weeks of age at the initiation of experiments. All mice were uniquely ear marked upon arrival to Monell to allow individual identification throughout the study.

#### Urine donor experimental treatment

Cages and individual mice within cages were randomly assigned to experimental treatments (Fig. 1A). To induce physiological and behavioral symptoms of sickness, we injected mice with lipopolysaccharide (LPS). LPS is an endotoxin, and an antigenic component in the cell wall of gram negative bacteria such as *Escherichia coli* (*E*. *coli*). Injection with LPS acutely reduces physical and social activity levels, induces lethargy, reduced feeding and drinking behavior, fever, and body mass loss^23,41,43^, but animals typically return to starting body mass and increase activity levels within several days. Because it is not a live replicating pathogen, LPS cannot be transmitted between or among individuals. We obtained LPS from *E*. *coli* as a lypholized powder from Sigma Aldrich Co. (product L2630, St. Louis, MO) and prepared LPS solution in 0.01 M sterile phosphate buffered saline (PBS) at a concentration of 0.20 mg/mL for intraperitoneal delivery of 200 uL (40 ug per mouse (ave body mass 20 g) or ~ 2 mg/Kg)). Mice injected with LPS were referred to as “sick”. Healthy urine donor animals received matching volume intraperitoneal injections of a 0.01 M sterile PBS solution. PBS does not induce inflammation or immune activation. Mice injected with PBS were referred to in two ways, depending solely on who they were cohoused with. Healthy PBS-injected mice cohoused with other healthy PBS-injected mice were referred to as “healthy”, while healthy PBS-injected mice cohoused with sick LPS-injected mice were referred to as “exposed” (Fig. 1A). Sick and healthy donor mice providing urine for training and validation Y-maze trials were cohoused with partners of the same experimental treatment (Fig. 1A). Healthy and exposed donor mice providing urine for Y-maze generalization trials were cohoused with partners of the same or different experimental treatments (Fig. 1A). Consistency in cohousing of all urine donors controlled for the potentially confounding effects of social/pair housing and experimental treatment. Six healthy/PBS-injected and six sick/LPS-injected donors provided urine for odor discrimination experimental training trials, an additional six healthy and six sick donors provided urine for behavioral odor discrimination validation trials, and a final six healthy and six exposed donors provided urine for behavioral odor discrimination generalization trials.

#### Urine donor collection procedure

Urine is a potent source of body odor in mice and also constitutes one component of scent marks used in territorial behavior^65–67^. Urine was directly collected from donor mice by light abdominal pressure. This is a minimally invasive and extremely rapid (<30 s) method that allows repeated sample collection from the same individual over time. This method of collection was especially valuable in this study, because it reduced the possibility of sample contamination from the housing environment or due to exposure of urine to air and microorganisms for long periods of time during the collection process. Investigators also changed gloves between sampling of mice from different treatment groups, further reducing the possibility of odor transfer in the process of urine collection from one sample to another. Donor animals quickly became acclimated to the direct urine collection procedure. Urine volume collected from each individual mouse varied from approximately 5 uL to ~80 uL per day; on some days there were individual donors that had empty bladders, and did not provide a sample. Collected urine was stored separately for each donor mouse and each day of collection and all samples were transferred to a −5 °C freezer immediately after collection. When we were ready to use urine samples in the behavioral odor discrimination assay trials, we chose 3 samples from the same mouse over several days ranging from 1–15 days post-LPS or post-PBS injection (e.g., urine samples collected 2, 4, and 8 days after injections for mouse # 1) and pooled these samples to obtain enough urine to be used as stimulus. In general, pooled urine samples were a volume of ~0.25 mL.

#### Biosensor mouse care and testing in the Y-maze

Ten mice were initially selected for behavioral odor discrimination bioassays. We called these animals “biosensor mice”, but they have also been referred to in previous studies as “sniffer mice”^19,50^. Biosensor mice were trained to perform the behavioral task of urine odor discrimination in the Y-maze assay. In this study, biosensor mice were adult (9–18 month) female C57BL/6 J mice, bred and maintained at the Monell Center. These mice initially underwent acclimation to the Y-maze odor discrimination testing apparatus at approximately 6 months of age, after which time they were exposed to training panels, consisting first of urine from two different mouse strains and later consisting of urine from sick and healthy mice. Initial biosensor mouse training for our specific experiment took place over a range of 7–15 days. Each day of training was carried out with a unique combination of sick and healthy urine donors. In later validation and generalization trials, biosensor mice were exposed to unique and completely novel pairs urine from donor mice. All biosensor mice were housed individually in a separate room from urine donors, but they were maintained under identical environmental and husbandry conditions as urine donor mice. All biosensor mice underwent water restriction for 23 h prior to Y-maze behavioral trials.

A Y-maze apparatus was used to test behavioral odor discrimination of biosensor mice as previously described^19,23,41,50^ (Supplementary Fig. S1). Air was continuously blown over two 35 mm Petri dishes containing approximately 0.25 mL urine from donor mice. Petri dishes containing urine were randomly assigned and enclosed on either side of the Y-maze “arms” and were inaccessible to physical contact by biosensor mice at all times. Air was blown from the ends of the two arms of the Y-maze through the neck of the maze, and down the central arm of the maze to a gated starting box area, where each biosensor mouse was placed at the start of each new trial. The start box gate was manually raised and lowered in a timed sequence, and two additional gates located at the entrances to the arms of the Y-maze allowed the biosensor mice to be contained after their odor/Y-maze arm choice was made. A positive response to one of the odors in the Y-maze was determined when biosensor mice ceased olfactory scanning activity, or odor tracking, through the Y-maze and displayed characteristic digging and scratching behaviors toward the closed reward box adjacent to the volatilized odor source. Trained biosensor mice often made the decision between the two arms of the Y-maze within 1–3 seconds of opening the start box. During training trials, biosensor mice were rewarded for a correct choice of odor by investigator removal of the metal cover containing the water source (an open tipped conical tube), which allowed the mouse to retrieve a single drop of water. Conical tubes holding a single drop of water were placed on both sides of the Y-maze at all times. In training within the Y-maze, mice can be reinforced to either of the odors presented (i.e., odors of sick or healthy urine donors, in our experimental training paradigm). We made the *a priori* choice to always reinforce biosensor mouse training to the odors of sick mice because we were primarily interested in whether mice could attend to potentially similar odor changes in our generalization trials comparing healthy and exposed urine donor mice (Fig. 1A,B). In experimental extinction, validation, and generalization trials (described in further detail, below) biosensor mice did not receive a water reward, and were instead transferred immediately back to the Y-maze start box after their odor choice was made and recorded. On average, 48 individual trials were run by each biosensor mouse during a single test session, which included rewarded training trials, unrewarded training trials (extinctions), and validation or generalization trials spaced evenly throughout the session. Experimental validation and generalization sessions occurred once per week and were interspersed from week to week. A single session included validation or generalization trials, but never both. A single validation or generalization session typically consisted of 5–6 unrewarded trials with novel donors, 30–40 rewarded training trials, and 5–6 unrewarded training trials. Four different types of experimental trials were used in the Y-maze paradigm, described below.

- *Rewarded training trials*: Biosensors mice received a water reward for a “correct” choice of the odor box containing urine from a sick conspecific. To reduce the possibility that mice were being trained to individual identities rather than donor treatment, unique combinations of training donors were used throughout training. Identity of the samples was known to the operator of the Y-maze in training (sick versus healthy), so she could provide an immediate water reward when the correct choice was made. There were two types of training trials of interest. Initial, or primary rewarded training trials were completed until biosensor mice reached an average of 80% correct responses to sick mouse urine odors in two consecutive blocks of training trials. This average “success threshold” of 80% correct responses was set *a priori*, and mice were not permitted to continue in further trials if they performed in training below this level. In our study, one of the ten biosensor mice did not successfully perform above the 80% success threshold and was eliminated from further trials. Rewarded *experimental* training trials were interspersed throughout experimental validation and generalization trials, such that mice were always exposed to rewarded and unrewarded trials within each testing session. The number of rewarded training trials within a validation or generalization trial ranged from 24–30.
- *Extinction/unrewarded training trials:* Extinction trials were introduced so that biosensor mice could gain exposure to unrewarded trials, which were subsequently used in validations and experimental generalization sessions. Intermittent partial reinforcement also serves to strengthen operant responses^68^. Unrewarded extinction trials were run with urine from sick and healthy training donors (i.e., urine samples were familiar to biosensor mice because they had been presented at least once before in training). Extinction trials were interspersed within validation and generalization sessions, and ~5 extinction trials for each individual biosensor mouse were run within in each testing session.
- *Validation trials:* Experimental Y-maze validation trials served as a type of generalization trial. Validation trials offered an additional level of assurance that biosensor mice were learning about treatment, rather than individual differences. In validation trials, we presented novel urine donor mice exposed to the same experimental treatments as training donors (sick versus healthy) to biosensor animals. Biosensor mice were exposed to these unique pairs only once across each validation session, but biosensor mice were typically presented with 5 validation trials per validation session. Investigators were blind to the identity of these samples (a second investigator recorded sample ID but did not run biosensor mice in any trials), and mice were unrewarded for their choice of odor/Y-maze arm. Performance in validation trials was scored at the end of experimental Y-maze behavioral assay.
- *Experimental generalization/test trials*: Experimental generalization trials allowed us to ask how trained biosensor mice generalized their learned responses to completely new conditions applied to another novel set urine donors (Fig. 1B). In other words, generalization trials provided a crucial test of our hypothesis. Generalization trials were designed to ask trained biosensor mice to compare the odors of healthy and exposed mice (Fig. 1A,B). As with experimental validation trials, investigators were blind to sample identity, samples were randomly assigned to Y-maze arms, and mice were unrewarded for their choice of odor/Y-maze arm. Performance in generalization/test trials was scored at the end of Y-maze behavioral assay (i.e., after trials were completed). As with extinction and validation trials, a total of 5 generalization trials were run within each generalization session.

#### Statistical analysis of biosensor responses in the Y-maze

Statistical procedures applied to the behavioral odor discrimination assays using the Y-maze setup were carried out in R Version 3.3.2^69^. Figures were made using R package ‘ggplot2’^70^. To compare performance in the behavioral odor discrimination Y-maze assay, we used an exact test of binomial proportion. This test examines whether the proportion of biosensor animal responses to one or the other arm of the Y-maze differs significantly from chance, or 50/50 choice of the left or right arm of the Y-maze^23^. Before carrying out the binomial test, we used a generalized linear model with a Poisson distribution (using the ‘glm’ function in R package ‘lme4’^71^) to test for main and interactive effects of time (day on which trials were carried out), biosensor mouse ID, and trial type (extinctions carried out within validation sessions versus extinction trials carried out within generalization sessions) on our outcome of interest (number of correct versus incorrect responses in Y-maze generalization trials). This allowed us to test whether mouse biosensors were behaving identically to unrewarded training samples from sick and healthy urine donors across sessions, and therefore allowed us to rule out the possibility that changes in individual biosensor mouse performance over time was confounded with our question of interest, about responses to novel donors in the experimental validation and generalization sessions. We used log total number of trials performed for each biosensor animal as an offset term in this predictive model. Because there were no significant main or interactive effects of time, biosensor mouse ID, or trial type, we pooled biosensor mouse responses across sessions, and by trial type. That is, performance for each biosensor animal was averaged across-validation sessions (e.g., 15–20 validation trials), across generalization sessions (e.g., 15–20 generalization trials), and extinction sessions (e.g., 30–40 extinction trials).

### Cohorts 2 and 3: Statistical predictive modeling based on urine odorants identified by GC-MS

#### Urine donor care

We examined two additional cohorts of mice (cohorts 2 (N = 56 mice) and 3 (N = 20)) in the subsequent portion of our study, designed to chemically discriminate urinary volatile profiles. Urine samples from donor mice in cohort 2 and 3 were subjected to headspace analysis employing gas-chromatography mass-spectrometry (GC-MS). As with cohort 1, urine donors in cohorts 2 and 3 were also obtained from Jackson Laboratories (Bar Harbor, ME), were approximately the same age and body size at the time of experiments as urine donors in cohort 1. Furthermore, donor mice providing urine samples for our chemical discrimination assays were treated identically to urine donors for the behavioral discrimination assays, in terms of their housing conditions, food and water availability, frequency of cage changes, and handling, as described above.

#### Urine donor experimental treatment

Experimental treatments randomly applied to individual urine donors in cohorts 2 and 3 were carried out identically to cohort 1. The single difference between urine donors in cohort 2 and 3 was in housing setup. Urine donor mice in cohort 2 were cohoused (one healthy mouse with another healthy mouse or one healthy mouse with a sick mouse), similarly to generalization donors in cohort 1) and allowed to freely interact and make contact with each other throughout the cages. Nine cages contained paired healthy mice (n = 18 healthy) and 19 cages contained paired sick and exposed mice (n = 19 sick and n = 19 exposed, Fig. 1A). Urine donor mice in cohort 3 were cohoused in cages of the same size and dimensions as cohort 2, but cages were divided with plastic, semi-permeable/perforated inserts, which permitted olfactory, auditory, and slightly obscured visual communication between sides of the cage (Supplementary Fig. S6). Each cage divider had 8, 0.5 mm holes at regularly spaced intervals. Dividers prevented bodily contact between mice and prevented contact with virtually all bedding and waste materials of animals on the adjacent side of the cage. Cages tops were also open to the environment, and therefore permitted air/volatile odorant transfer both above and through the cage partitions. In cohort 3, three cages contained paired healthy mice (n = 6 healthy) and 7 cages contained paired sick and exposed mice (n = 7 sick and n = 7 exposed, Fig. 1A).

#### Urine donor collection procedure

Urine collection procedures for donor mice in cohorts 2 and 3 were identical to those for cohort 1. Urine samples were never pooled for chemical analysis. At least 25 uL urine was required per sample to run chemical analysis.

#### Urine analysis procedures with GC-MS

Urine samples (25 uL) from individual donor mice were prepared for chemical analysis by fortification with an internal standard, 800 ng l-carvone. We added 10 uL of an aqueous fortification solution of carvone to mouse urine. Vials containing no urine samples (carvone blanks) were also fortified with 10 uL of the carvone solution and analyzed by GC-MS. Samples were subjected to headspace analysis using a HT3 dynamic headspace analyzer (Teledyne Tekmar, Mason, OH, USA) outfitted with Supelco Trap K Vocarb 3000 trap (Sigma-Aldrich Co., St. Louis, MO) and as described elsewhere^24^ using splitless injection. Briefly, during headspace collection, samples were maintained at 40 °C, swept with helium for 10 minutes (flow rate of 75 mL/min), and the volatiles collected directly on the thermal desorption trap. Trap contents were desorbed at 260 °C directly into a Thermal Scientific ISQ single-quadropole gas chromatograph-mass spectrometer (GC-MS; Thermo Scientific) equipped with a 30 m × 0.25 mm id Stabiliwax-DA fused-silica capillary column (Restek). The GC oven program consisted of an initial temperature of 40 °C (held for 3 min) followed by a ramp of 7 °C/min to a final temperature of 230 °C (held for 6 min). The mass spectrometer was operated in scan mode from 33 to 400 m/z. Volatile compound identification was determined based on spectral library matching in the NIST 528 Standard Reference Database (Supplementary Table S1). For known mouse urine volatiles with poor spectral library matches (e.g.,<50% matches), we examined relative retention times among known mouse urine volatiles on Stabilwax columns to assist with compound identification^51–53^. Baseline correction, noise elimination, and peak alignment of the chromatographic data were performed with Metalign^72^ and the MSClust tool was used to perform mass spectral extraction for generation of selected ion chormatographic peak responses^73^.

We averaged peak responses over two samples for each individual, consisting of urine collected between days 4 and 15 post treatment exposure. For all mice, a single sample within the range of days 4–7 and another sample within the range of days 9–15 were analyzed. In several cases, we did not collect enough urine volume for mice, and so, these animals were omitted from our analysis (e.g., starting sample size was not identical to sample size in analyses). Urine samples for both cohorts of mice were run interspersed throughout our GC/MS runs to avoid the potentially confounding effect of time on instrumentation results.

#### Statistical predictive modeling of odorants described by GC-MS

We analyzed chemical data from the two cohorts of mice (cohort 2 and 3) separately, but the processes of model/feature selection from an initially larger number of all possible identified chromatographic peaks, followed by linear discriminant analysis and cross-validation performed on the training data set, and subsequent classification-based predictions on a novel test data set were identical (and similar to previously published methods^24^). We performed stepwise model selection using the package ‘klaR’^74^ on the training data set, consisting of healthy and sick mouse samples, using the function ‘greedy.wilks’. We stopped at inclusion or exclusion of peaks in the stepwise selection process when the squared canonical correlation value was greater than or equal to at least 0.60 (or 60%). We set this threshold for model selection *a priori*, as previous experience with similar data sets suggested that this threshold of 0.60 would likely be adequate for desired classification performance of the model (*e*.*g*., to minimize the bias-variance tradeoff) in cross-validation and subsequent predictions on the test data set. We applied a linear discriminant analysis with the ‘lda’ function in R package ‘car’^75^ and used leave-one-out cross-validation to assess the accuracy of our model, fit to the training data set. Finally, we used the ‘predict’ function to perform predictive classification of our test data set, which included exposed mice in cohorts 2 and 3 (analyzed separately). The test data set was not used in initial model building/feature selection. We present results as the number of exposed mice in the test data set classified as sick versus the number of exposed mice classified as healthy. To statistically compare the difference in classification of exposed mouse samples as sick versus healthy, we used a two-tailed non-parametric Wilcoxon rank sum test with continuity correction. This statistical test was performed using the posterior probability scores for classifying each individual test observation as sick or healthy. Posterior probability scores represent the degree of certainty (expressed as a percentage) with which each test sample was predictively classified as sick versus healthy). The Wilcoxon test examined the hypothesis that the mean posterior probabilities were equal for sick versus healthy classification. Thus, a significant P value suggests a significant difference in the average probability of exposed mice being classified as sick versus healthy. Although it was not the intent of this study to make individual pairwise comparisons of individual compounds identified using model selection techniques, we present suppelmentary results of pairwise two-sample t tests (or a non-parametric alternative), comparing compound levels between sick and healthy mice, in Supplementary Table S2A,B.

## Electronic supplementary material

Supplementary Information

## Data Availability

All data are available in Dryad.
