# Supplementary material for: Sharing an environment with sick conspecifics alters odors of healthy animals
Source: Sci Rep. 2018 Sep 24;8:14255. doi: 10.1038/s41598-018-32619-4 (PMC6155122; doi:10.1038/s41598-018-32619-4)
Supplement: Supplementary file 1 — Supplementary Information [file 41598_2018_32619_MOESM1_ESM.pdf]

TITLE PAGE – SUPPLEMENTARY INFORMATION

Title: Sharing an environment with sick conspecifics alters odors of healthy animals

Authors: Stephanie S. Gervasi[1], Maryanne Opiekun[1], Talia Martin[1,] Gary K. Beauchamp[1] and Bruce A. Kimball[1,2]

Author Affiliations:

[1]Monell Chemical Senses Center, 3500 Market Street, Philadelphia, PA 19104, USA, [2]USDA-APHIS-WS-NWRC

Correspondence to be sent to: Stephanie S. Gervasi, Monell Chemical Senses Center, 3500 Market Street, Philadelphia, PA 19104, USA. e-mail: sgervasi@monell.org

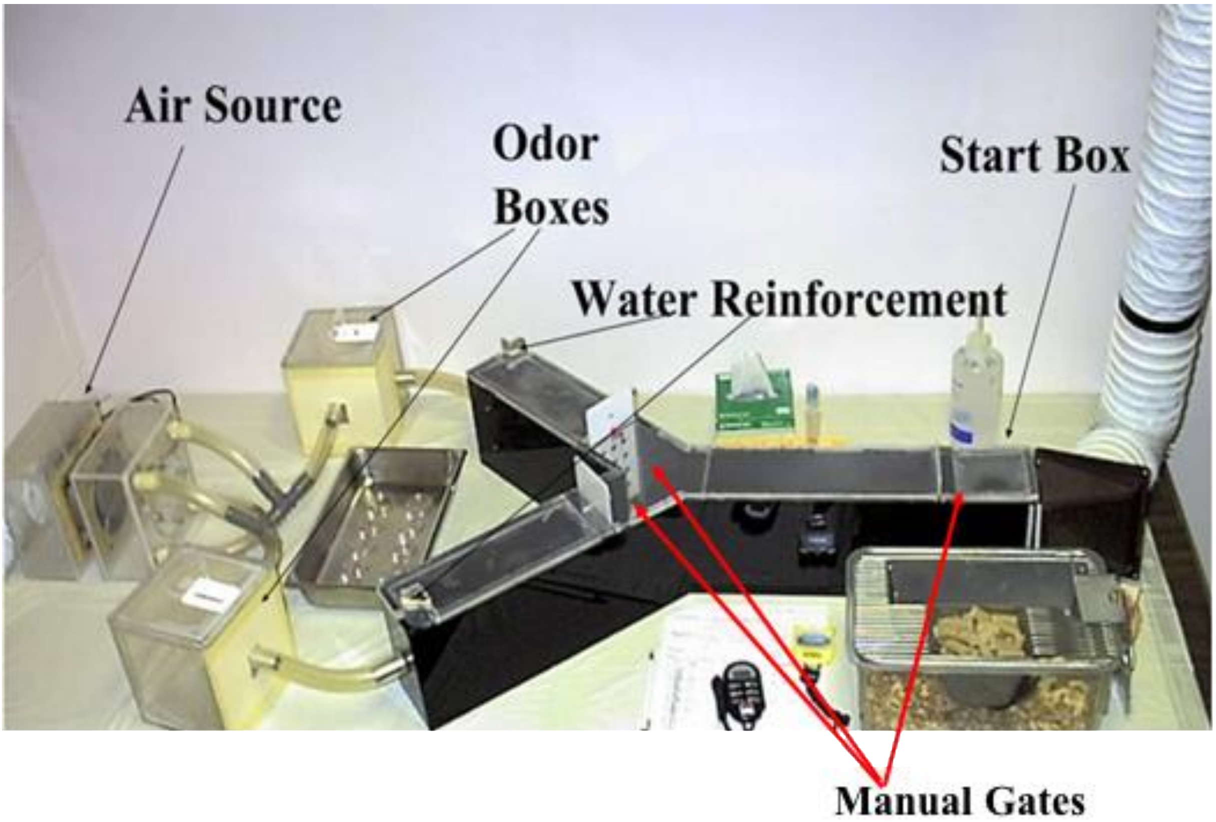

Supplementary Figure S1. Overview of the Y-maze Setup. The Y-maze behavioral odor discrimination learning paradigm allows investigators to train mice via positive reinforcement to differentiate volatile odors from two sources. Following training, non-reinforced, blind, experimental generalization trials allow investigators to ask about similarities in odors arising from different manipulations. In the Y-maze setup, the two distal arms are connected to odor boxes containing an open source of the odor (*e.g.*, urine held in plastic Petri dishes). An air source, or fan, sends air in a single direction through the odor boxes and into the distal arms of the Y-maze. Mice perceive odors as they move down the stem of the Y-maze from the start box. A gate at the exit of the start box contains biosensor mice before the trial. Gates at the entrance to each of the Y-maze arms contain mice after they have made a choice of odor. Trials are timed, and the Y-maze is cleaned between trials.

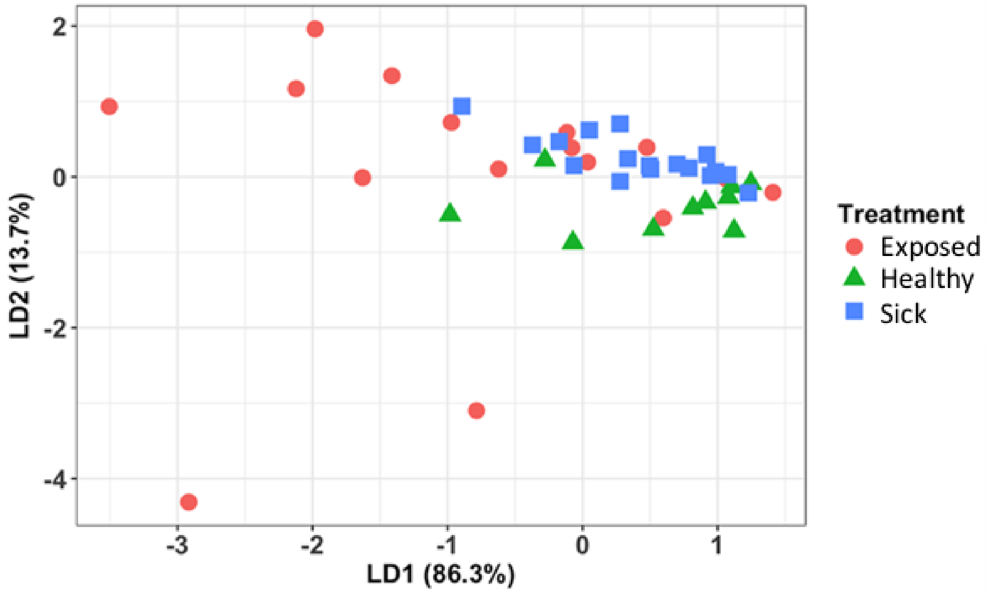

Supplementary Figure S2. Linear discriminant analysis (LDA) plot showing grouping of samples across treatments in the cohoused and freely interacting mice (cohort 2). The LDA model included 3 explanatory chromatographic peaks representing different urinary volatiles and was selected and cross-validated on a training data set of urine samples from sick and healthy mice. Classification predictions based on this LDA model and applied to our test set of exposed mice classified 12/16 of exposed mice as sick and 4/16 of exposed mice as healthy. Sample sizes reflected in the figure are different from original starting sample sizes for cohort 2 due to lack of urine samples for several animals.

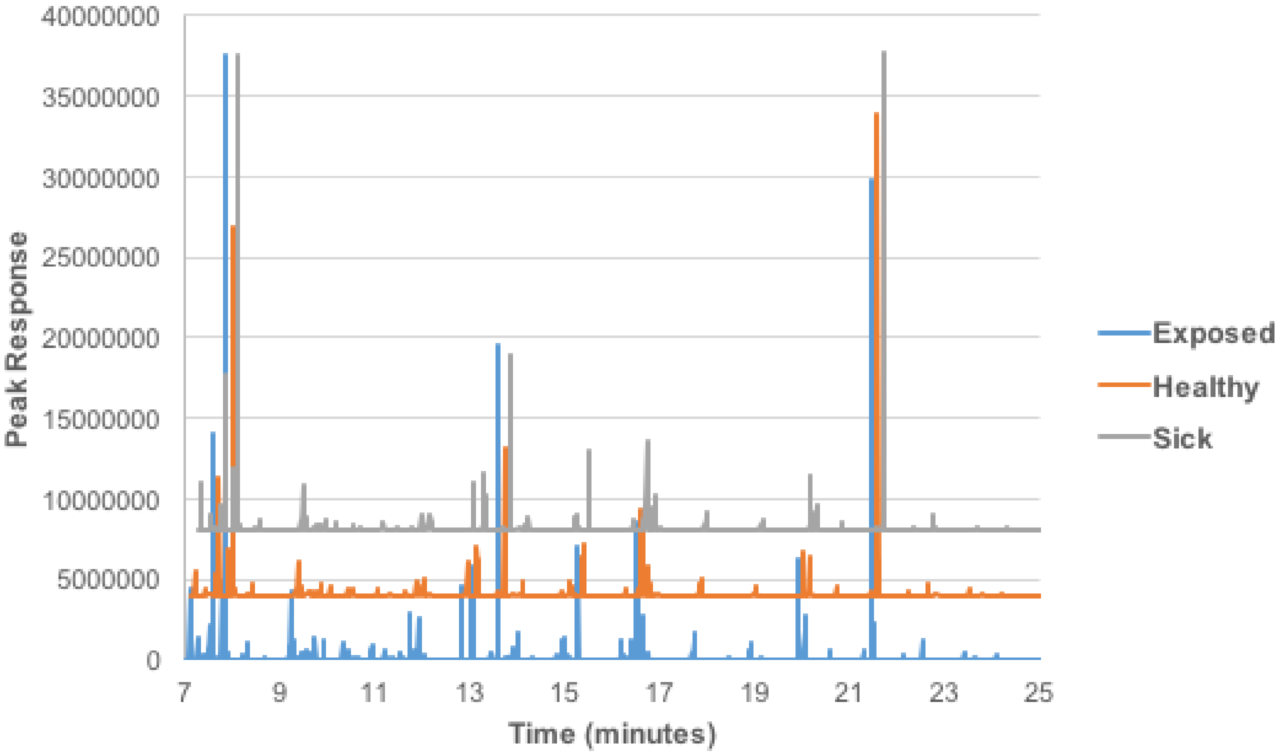

Supplementary Figure S3. **Mean processed chromatograms of urinary volatiles for the cohoused and freely interacting mice (cohort 2)**. Chromatograms are offset horizontally and vertically to allow direct comparison of peak responses across retention times (in minutes) for exposed, healthy, and sick mice. Chromatographic peak responses have undergone peak alignment and noise reduction in Metalign and all peak responses were normalized to an internal standard (carvone) peak. Peaks selected in linear discriminant models appear at scan retention times of 14.79 (2-acetyl-2-thiazoline), 16.98 (decanal), and 24.05 (dimethyl sulfone). Carvone had a retention time of 21.4.

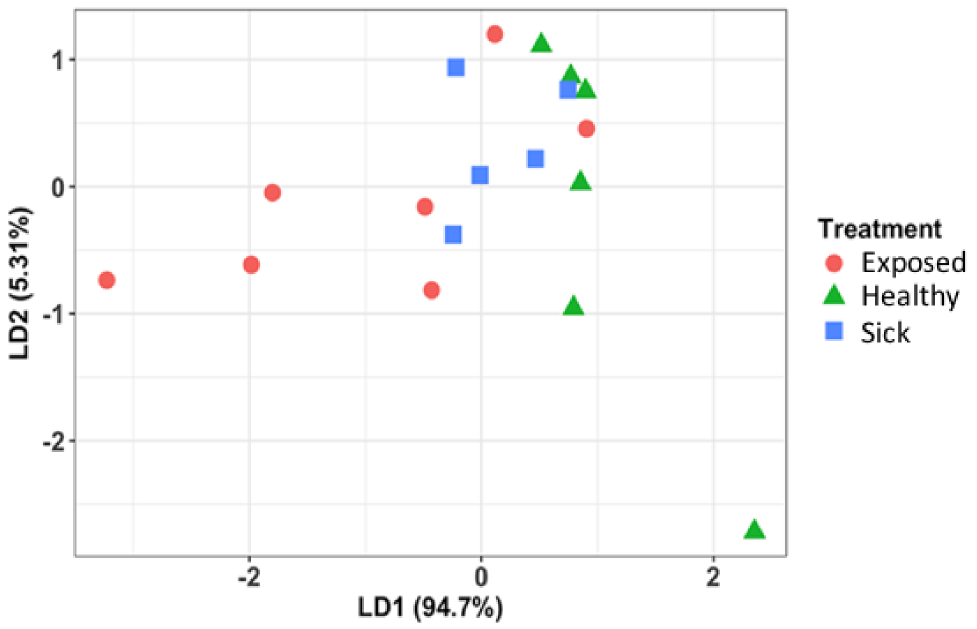

Supplementary Figure S4. Linear discriminant analysis (LDA) plot showing grouping of samples across treatments in the cohoused but physically separated mice (cohort 3). The LDA model included 3 explanatory chromatographic peaks representing different urinary volatiles and was selected and cross-validated on a training data set of urine samples from sick and healthy mice. Classification predictions based on this LDA model and applied to our test set of exposed mice classified 6/7 exposed mice as sick and 1/7 of exposed mice as healthy. Sample sizes reflected in the figure are different from original starting sample sizes for cohort 3 due to lack of urine samples for several animals.

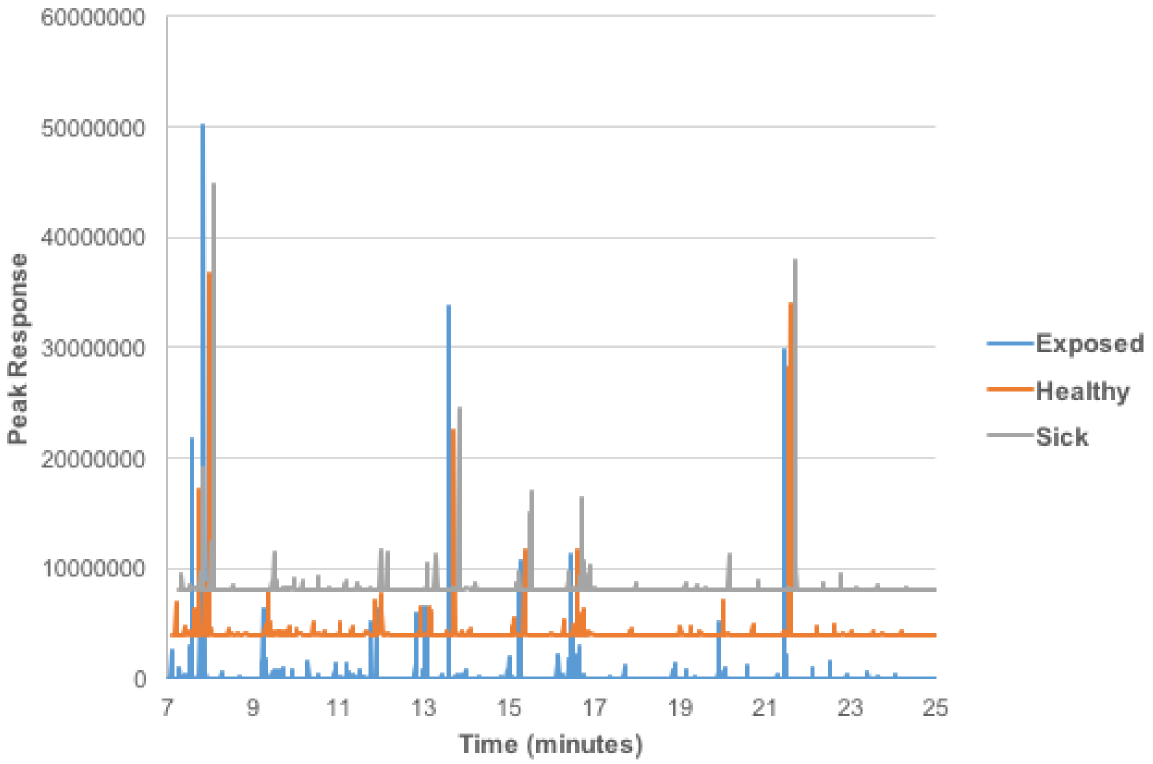

Supplementary Figure S5. **Mean processed chromatograms of urinary volatiles for the cohoused but physically separated mice (cohort 3)**. Chromatograms are offset horizontally and vertically to allow direct comparison of peak responses across retention times (in minutes) for exposed, healthy, and sick mice. Chromatographic peak responses have undergone peak alignment and noise reduction in Metalign and all peak responses were normalized to an internal standard (carvone) peak. Peaks selected in linear discriminant models appear at scan retention times of 10.97 (6-methyl-3-heptanone), 13.01 (4-methyl-6-hepten-3-one), and 15.23 (dehydro-exo-brevicomin). Carvone had a retention time of 21.4.

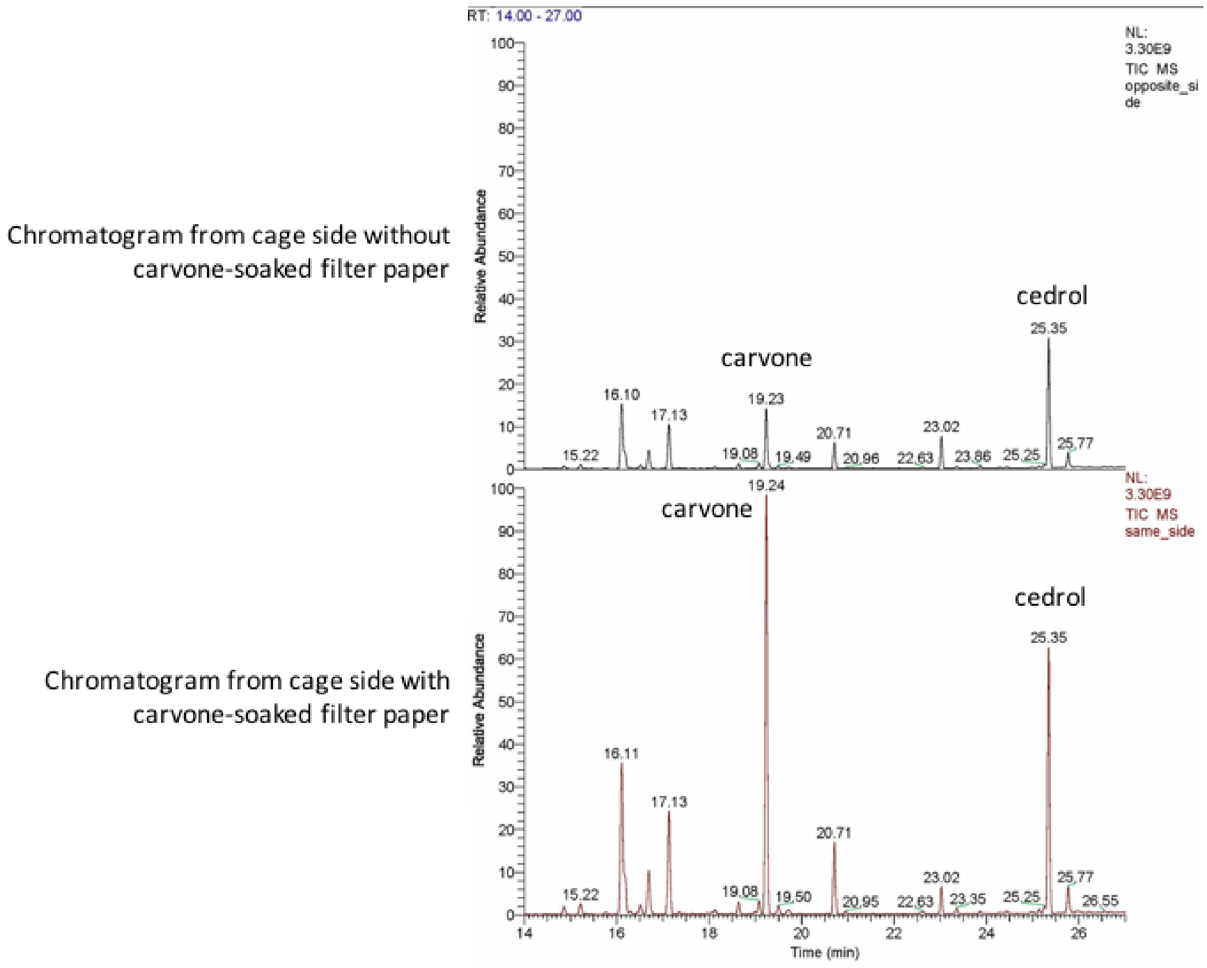

Supplementary Figure S6. **Confirmation of volatile odorant transfer in partitioned cages used in cohort 3.** Confirmation of odorant transfer was conducted by monitoring volatiles on both sides of a cage separated by semi-permanent partitions with small holes permitting odorant transfer throughout the cage but precluding contact between animals. Twenty-five uL of neat carvone (99% L (1) - carvone CAS 6485-40-1, Sigma Aldrich Co., St. Louis, MO) was added to a filter paper placed on one side of the partitioned cage set up with bedding, partitions, food, and water, identically to experimental conditions, but without mice. We allowed carvone to equilibrate and the volatiles to migrate across sides of the cage for 90 minutes. Volatile carvone was monitored on both sides of the cage by solid phase micro-extraction (SPME) and gas chromatography-mass spectrometry (GC/MS). A divinylbenzene/carboxen/PDMS fiber

(Supelco, Sigma-Aldrich, St. Louis, MO) was sequentially exposed for 10 minutes (placed approximately 5 cm above the bedding) to the two sides of the cage (starting with the side of the cage opposite of the carvone filter paper) and desorbed in a GC/MS using similar parameters as used for urine analyses. Peak area responses were determined for carvone and cedrol (representing bedding volatiles) and carvone/cedrol ratios were determined for both sides of the cage. The ratio was 0.50 on the side of the cage opposite from carvone and 1.65 on the side of the cage where carvone was placed. Therefore, we estimated a 30% transfer of the carvone volatiles across the partitioned cage in a 90-minute period.

Supplementary Table S1. Volatile compound identification based on spectral library matching in the NIST 528 Standard Reference Database. For known mouse urine volatiles with poor spectral library matches (e.g., < 50% matching) we also used relative retention times from previously published studies for urine volatiles on Stabilwax columns to assist with compound identification[24,52-54].

Percent Matching	Compound ID
94.12	Dimethyl sulfone
80.80	2-acetyl-2-thiazoline
67.72	Dehydro-exo-brevicomin
47.47	6-methyl-3-heptanone
36.30	Decanal
33.11	4-methyl-6-hepten-3-one

Supplementary Table S2A. Standardized peak responses fort the top explanatory compounds chosen by stepwise selection techniques to discriminate between sick (LPS-injected) and healthy (PBS-injected) mice in cohort 2 (freely-interacting pairs). Both sick and healthy mice were cohoused with healthy (PBS-injected) conspecifics. Presented below are the peak responses as well as the mean peak response for each group. Below, in blue, we also present the test statistic values from a two-sample t-test (t value) or a Wilcoxon rank test (W value) and P values associated with the parametric or non-parametric pairwise comparisons of peak responses, respectively. A Shapiro-Wilk test was used to test for normal distribution of values in the treatment groups before performing pairwise comparisons. If data met the assumption of normality, we then performed an F-test for homogeneity of variance. If variance was not equal between the two groups, we used the Welch two sample t-test. All tests were two-tailed. This pairwise comparison approach was not taken in our primary analysis presented in the paper. Instead, we report on our *a priori* planned pattern recognition approach using stepwise model selection and linear discriminant analysis. We present this table of pairwise comparisons for additional information to our readers. Significance of the pairwise comparison for individual compounds at an alpha level ≤ 0.05 is denoted with an asterisk. Marginal significance at an alpha level ≤ 0.1 is denoted with two asterisks.

cohort	mouseid	treatment	Peak35/2-acetyl-2-thiazoline	Peak46/decanal	Peak64/dimethyl sulfone
2	1	PBS	0.01246318	0.000424674	0.0049244
2	3	PBS	0.025115763	0.002463231	0.014713227
2	4	PBS	0.004775315	0.000108377	0.00847129
2	5	PBS	0.002870948	0.000173318	0.007212274
2	6	PBS	0.006697177	0.000858409	0.007448176
2	10	PBS	0.029152277	0.000682354	0.026998519
2	11	PBS	0.011160152	0.000527674	0.009557259
2	15	PBS	0.01249618	0.001284187	0.023484772
2	17	PBS	0.009414021	8.16E-05	0.009297405
2	18	PBS	0.017683077	0.000744739	0.01081114
		mean	***0.01318***	***0.0007349***	***0.0123***
2	20	LPS	0.007682909	0.002787691	0.02275812
2	22	LPS	0.003099849	0.000158579	0.010718528
2	24	LPS	0.004316406	0.000373439	0.022933472
2	26	LPS	0.004029321	0.001145564	0.012300116
2	28	LPS	0.008833016	0.000233208	0.034207858
2	30	LPS	0.006640277	0.000268178	0.015965481
2	32	LPS	0.003918306	0.002745997	0.005045713
2	34	LPS	0.00413022	0.001216024	0.013416885
2	36	LPS	0.00048386	0.002203794	0.019880299
2	38	LPS	0.004226833	0.000129422	0.010823093
2	40	LPS	0.010469844	0.004555185	0.018771635
2	44	LPS	0.001956372	0.003000776	0.007683192
2	46	LPS	0.011050025	0.001643446	0.016117659
2	48	LPS	0.006774352	0.001554544	0.014796472
2	52	LPS	0.000132221	0.001879963	0.011430109
2	54	LPS	0.010715496	0.000974112	0.025700205
2	56	LPS	0.006865444	0.000116884	0.018180809
		mean	***0.005607***	***0.00146***	***0.01651***
		test statistic	***t = 2.683***	***W = 55***	***W = 49***
		p value	**0.0217***	**0.1406**	**0.0743****

Supplementary Table S2B. Standardized peak responses fort the top explanatory compounds chosen by stepwise selection techniques to discriminate between sick (LPS-injected) and healthy (PBS-injected) mice in cohort 3 (physically separated mice). Both sick and healthy mice were cohoused with healthy (PBS-injected) conspecifics. Presented below are the peak responses as well as the mean peak response for each group. Below, in blue, we also present the test statistic values from a two-sample t-test (t value) or a Wilcoxon rank test (W value) and P values associated with the parametric or non-parametric pairwise comparisons of peak responses, respectively. A Shapiro-Wilk test was used to test for normal distribution of values in the treatment groups before performing pairwise comparisons. If data met the assumption of normality, we then performed an F-test for homogeneity of variance. If variance was not equal between the two groups, we used the Welch two sample t-test. All tests were two-tailed. This pairwise comparison approach was not taken in our primary analysis presented in the paper. Instead, we report on our *a priori* planned pattern recognition approach using stepwise model selection and linear discriminant analysis. We present this table of pairwise comparisons for additional information to our readers. Significance of the pairwise comparison for individual compounds at an alpha level ≤ 0.05 is denoted with an asterisk. Marginal significance at an alpha level ≤ 0.1 is denoted with two asterisks.

cohort	mouseid	treatment	Peak16/6-methyl-3-heptanone	Peak24/4-methyl-6-hepten-3-one	Peak37/dehydro-*exo* -brevicomin
3	1	PBS	0.00010006	0.02229561	0.22530877
3	2	PBS	0.00035541	0.11341283	0.68533675
3	3	PBS	0.00008600	0.03431548	0.22722250
3	4	PBS	0.00048390	0.09324524	0.09959502
3	5	PBS	0.00008080	0.02814950	0.39939568
3	6	PBS	0.00021799	0.05009372	0.48532333
		mean	***0.000221***	***0.0569***	***0.3537***
3	8	LPS	0.0001105	0.1488467	0.5370131
3	12	LPS	0.0002622	0.2602205	0.5834637
3	14	LPS	0.0002032	0.1909025	0.5129344
3	16	LPS	0.0001668	0.1283553	0.3199192
3	20	LPS	0.0000728	0.0743114	0.0651735
		mean	***0.000163***	***0.161***	***0.4037***
		test statistic	***t = 0.7088***	***t = -3.1496***	***t = -0.3869***
		p value	***0.4964***	***0.0117****	***0.7078***

Supplementary Table S3. Summary of within- and across- cohort model performance. Within-cohort model performance was assessed using leave-one-out cross validation, performed on the training data set consisting of urine samples from sick/LPS-injected and healthy/PBS-injected mice. Across-cohort model evaluation involved classification of healthy/PBS-injected or sick/LPS-injected mouse samples from one cohort based on features/peaks selected to discriminate between sick and healthy mouse samples in the other cohort (e.g., cohort 2 model applied to sick and healthy mice in cohort 3, or vice versa). Because across-cohort models performed poorly, we made all hypothesis-driven predictions about exposed mice (i.e., healthy mice housed with sick conspecifics) within cohorts.

	**# of sick/LPS-injected mice classified as sick/LPS- injected** (*sensitivity*)	**# of healthy/PBS-injected mice classified as healthy/PBS-injected *(specificity)***
**Within-cohort model performance**		
Cohort 2 model	17/17 (100%)	7/10 (70%)
Cohort 3 model	5/5 (100%)	6/6 (100%)
**Across-cohort model performance**		
Cohort 2 model applied to cohort 3	2/5 (40%)	2/6 (33%)
Cohort 3 model applied to cohort 2	13/17(76%)	3/10 (30%)

206
